# The Use of Thermal Techniques for the Characterization and Selection of Natural Biomaterials

**DOI:** 10.3390/jfb2030230

**Published:** 2011-09-13

**Authors:** Valérie Samouillan, Florian Delaunay, Jany Dandurand, Nofel Merbahi, Jean-Pierre Gardou, Mohammed Yousfi, Alessandro Gandaglia, Michel Spina, Colette Lacabanne

**Affiliations:** 1 Physique des Polymères, Institut Carnot CIRIMAT UMR5085, Université Paul Sabatier, 118 route de Narbonne, Bat 3R1B2, 31062 Toulouse Cedex, France; 2 LAPLACE, UMR CNRS 5213, Université Paul Sabatier, 118 Route de Narbonne, 31062 Toulouse Cedex 9, France; 3 Dipartimento di Scienze Biomediche Sperimentali, Università di Padova, Viale Colombo 3, 35131 Padova, Italy

**Keywords:** elastin glass transition, collagen denaturation, differential scanning calorimetry, thermal stability

## Abstract

In this paper we explore the ability of thermal analysis to check elastin and collagen integrity in different biomaterial applications. Differential Scanning Calorimetry (DSC) has been used to analyze the first and second order transitions of the biological macromolecules in the hydrated and dehydrated state. First, we report the characterization of control cardiovascular tissues such as pericardium, aortic wall and valvular leaflet. Their thermal properties are compared to pure elastin and pure collagen. Second, we present results obtained on two collagen rich tissues: pericardia with different chemical treatments and collagen with physical treatments. Finally, more complex cardiovascular tissues composed of elastin and collagen are analyzed and the effect of detergent treatment on the physical structure of collagen and elastin is brought to the fore.

## 1. Introduction

Development of biomaterials used as substitutes of the extracellular matrix for the replacement of cardiovascular tissues in associated pathologies (atherosclerosis, aneurysms and cardiac valvulopathies) or the realization of skin substitutes is a great challenge for repair medicine. Bioprosthesis conceiving from exogenic animal tissues is a promising issue to realize materials that mimic the mechanical properties of original tissues, allowing a repopulation by patients' own cells [1,2,3].

For two decades, detergent and enzymatic protocols have been optimized to obtain non antigenic extracellular matrices, preserving the main fibrillar proteins such as collagen, responsible for mechanical strength and elastin, responsible for elasticity [4,5,6]. Nevertheless, the durability of such bioprostheses must be improved by the mechanical stabilization of fibrillar proteins and their preserving against proteolytic degradation is necessary to a complete recellularization [7,8].

The failure of the main materials results from a combination of degradation, calcification or inappropriate tissue overgrowth [9,10]. The mechanism of calcification is still unclear, but seems to be associated with both cell membrane remnants and glutaraldehyde treatment. Indeed this treatment, thought to reduce immunogenicity, ensures survival of the implant by stabilizing the xenogeneic tissue against protein degradation [11], by a cross-linking action, as well as protein denaturation induced by anticalcification treatments [12]. However, it can also induce cytotoxicity [13] and change the material mechanical properties [14,15].

The present work is devoted the characterization of natural biomaterials for cardiovascular applications by the use of Differential Scanning Calorimetry (DSC). In this paper we chose to explore the ability of this calorimetric technique to extract thermal properties of collagen and elastin, and to check the integrity of these proteins in different biomaterials. The rich chemistry of collagen allows engineers to alter physical and chemical properties such as porosity, crystallinity and cross-links density. These *in vitro* characteristics allow controlled interaction with the host resulting in predictable tissue ingrowth and biodegradable rates [16]. The two first applications deal with chemical or physical modifications of collagen. The third application is devoted to the evaluation of structural modifications and possible damage of elastin and collagen in detergent treated aortic tissues.

## 2. Results and Discussion

### 2.1. Thermal Properties of Cardiovascular Tissues and Main Proteins

In this first section, we report the thermal properties of three cardiovascular tissues widely used in medical applications: bovine pericardium, consisting mainly of collagen type I, which is a relatively simple and uniform tissue, porcine valvular leaflet and porcine aortic walls, which are more complex tissues, typically fiber reinforced composite materials [17]. In the aortic wall and valvular leaflet, the elastin content accounted for about 60% and 6% of the total dry weight while the corresponding values for collagen were 23% and 70%, respectively [18]. In order to specify the origin of the thermal transitions of the three tissues, the characterization of elastin (purified from bovine nuchae ligamentum) and collagen type I (extracted from Bovine Achille's tendon) was performed.

#### 2.1.1. Characterization in the Hydrated State

**Figure 1:**
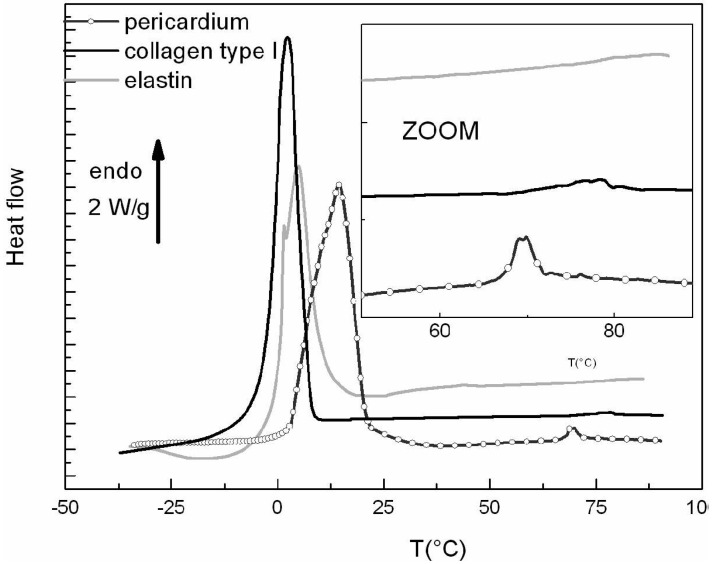
Differential Scanning Calorimetry (DSC) thermograms of bovine pericardium, collagen type I and elastin in the hydrated state recorded at 10 °C/min between −40 °C and 90 °C.

Figure 1 displays the DSC thermograms (first scans) of fresh bovine pericardium, hydrated elastin and hydrated type I collagen.

The melting of ice, corresponding to freezable water, is observed as a large endothermic peak between −5 to 20 °C according to the tissue. This extrinsic transition is widely used to quantify the amount of freezable water in hydrated proteins and tissues (by dividing the area of the measured endothermic peak by 330 J/g, corresponding to the melting enthalpy of pure ice), and completes the thermogravimetric analyses that give access to the total amount of water. For the three samples, the total hydration level was shown to be superior to 1 g/g of dry protein. By a simple difference, the amount of unfreezable water—namely bound water can be calculated and was found equal to 40% (by weight of dry sample) in collagen and pericardium, and 35% in elastin. These values, which correspond to the filling of the first hydration shell of the proteins, are close to literature data for a wide class of proteins [19]; as elastin contains a large part of apolar residues, its ability to fix water is reduced when compared to collagen. The enlargement of the 60–80 °C range highlights the presence of a second endothermic phenomenon for collagen and pericardium, associated with the denaturation phenomenon of collagen. It is well-known that a characteristic feature of collagen is the triple helical structure of three left-handed polyproline type helices twisted into a right-handed superhelix. The formation of such a structure is due to the repeating sequence Gly-X-Y, where X and Y are often proline and hydroxyproline, respectively, and hydrogen bonding takes place between chains within the triple helix. On heating, the triple helix unfolds to produce random chains of gelatin [20], that can remain covalently linked to each other or not depending on the degree of heating [21,22]. The denaturation phenomenon -distinct from degradation- implies that the rupture of interchain hydrogen bonds leads to the formation of an amorphous polymer, called gelatin. The denaturation parameters that we found were hydrated collagen type I (Td = 78.3 °C and ΔHd = 47.8 J/g. These are close to the thermal parameters of fully hydrated rat tail tendon reported in the literature [23]: Td = 65.1 °C and ΔHd = 58.55 J/g. The high enthalpy of unfolding collagen immersed in water is thought to derive mainly from the breaking of hydrogen bonds forming the hydration network around the collagen molecules [24,25,26]. The hydrogen bonding may be dominated by the number and layout of the fixed hydrogen bonding sites on the collagen itself, e.g., C=O, N–H, and hydroxyl groups on hydroxy-proline, that are exposed to the solvent and available for supporting hydrogen-bonded solvent bridges [24,25,26,27]. The thermogram of bovine pericardium, a collagen rich tissue, presents some analogies with the collagen type I thermogram previously analyzed. The denaturation temperature of collagen in the pericardium is found at 69.8 °C, and the associated enthalpy is 61.7 J/g, as roughly observed in literature on cardiovascular tissues [4]. The differences observed between the denaturation parameters of bovine pericardium and collagen type I extracted from a bovine tendon can be explained by the differences in collagen type (in pericardium coexist type I and type III collagens), by the purity of the tissue (elastin and glycoaminoglycans are conserved in pericardium), and by the different packing of fibers inducing entropic effects.

In contrast with collagen and bovine pericardium, the thermogram of elastin does not present any intrinsic endothermic peak; the only endothermic peak observed is associated with the melting of ice at around 0 °C. Contrary to collagen, elastin does not possess a long-range order, and although different secondary conformations in this protein, it can be considered as amorphous for the physical structure, which is in good agreement with the Tamburro's model on labile, dynamic β turns in hydrophobic domains [28] and the Daggett's model [29] that describes hydrophobic domains of elastin as compact amorphous structures. According to literature data, the only transition detectable for elastin is a glass transition, namely a pseudo-second order transition associated with a jump of the specific heat in DSC thermograms and due to the transition from a glassy to a rubber state [30,31,32,33]. We showed in a previous work [34] that water acts as a strong plasticizer for elastin, lowering the glass transition temperature Tg in a spectacular manner from 200 °C, in the dehydrated state, to the room temperature for a 30% hydration level. For higher hydration levels (>40%) the existence of a minimal glass transition temperature has been evidenced at 0 °C, when elastin fibers contain freezable water. In this case, the motions of some tens of nanometers along the polypeptidic chains are blocked until the beginning of ice melting. So in this case, the specific step of the glass transition is superimposed on the endothermic peak of ice melting as showed in the Figure 1.

#### 2.1.2. Characterization in the Freeze-Dried State

**Figure 2:**
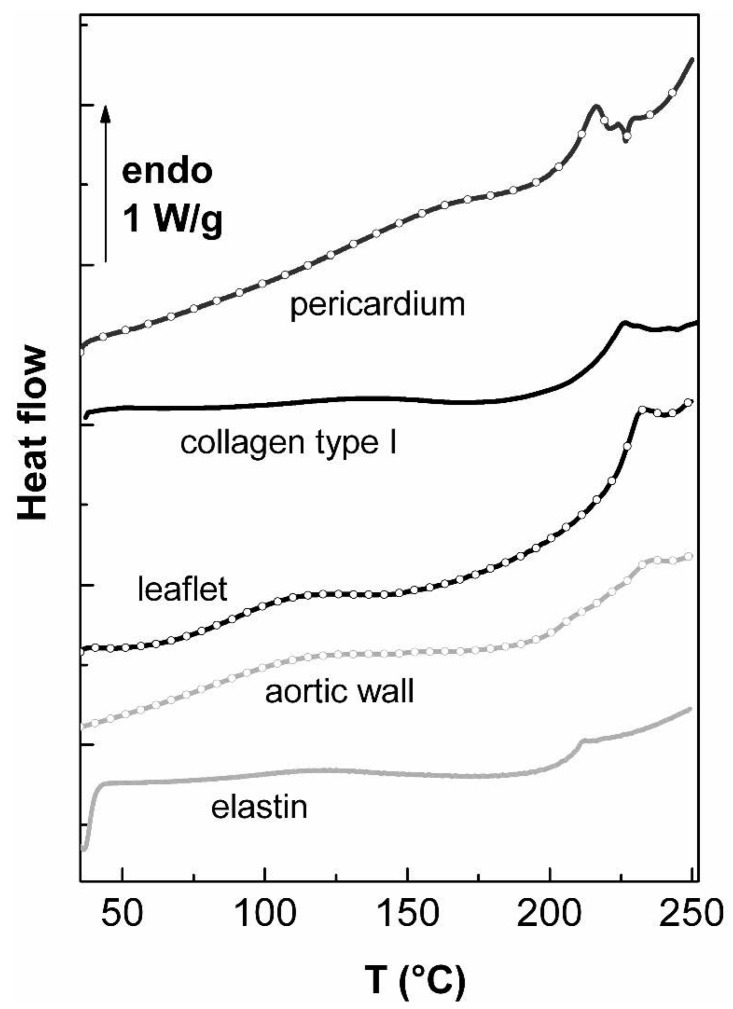
DSC thermograms of bovine pericardium, porcine valvular leaflet, porcine aortic wall, collagen type I and elastin in the dehydrated state recorded at 20 °C/min between 40 and 250 °C after a first scan performed between 30 and 150 °C.

Figure 2 shows the thermograms concerning the second scans of freeze-dried tissues (pericardium, aortic leaflet and aortic valve), collagen type I and elastin.

The first scan performed between 40 and 150 °C is not shown, since it is only characterized by a broad endothermic peak around 100 °C associated with water bound departure for all the samples [18]. In the second scans reported here intrinsic transitions can be observed for all the samples. For collagen type I, the endothermic peak of denaturation is observed for Td = 225 °C, with an enthalpy area ΔHd = 7.05 J/g. These values, very distinct from what found in the hydrated state, are similar to thermal parameters of pure freeze-dried collagen generally observed in literature [23]. In this case, the value of the denaturation enthalpy of collagen for hydration <6% (corresponding to less than one mole of water per tripeptide) is assigned mainly to the breaking of the direct hydrogen bonds between alpha chains. This endothermic peak, that can be assigned as a first order transition is not reversible on successive scans in the dry state. In this case, only a jump of the specific heat is observed around 200 °C, which is attributed to the glass transition of denatured collagen (*i.e.*, gelatin), characteristic of its amorphous nature [18].

For elastin, a jump of the specific heat is observed at 200 °C, as previously observed in the literature [33]. This pseudo-second order corresponds to the glass transition of the dry protein. As for collagen denaturation, there is a drastic shift toward high temperature of the glass transition, due to the replacement of hydrogen bonds between the polypeptidic chain and water by hydrogen bonds between polypeptidic chains without water bridges, associated with brittle and stiffer elastin.

The thermogram of freeze-dried bovine pericardium presents strong analogy with the pure collagen thermogram. The denaturation phenomenon is shifted toward high temperature when compared with fresh tissue, with Td = 214 °C and ΔHd = 12.3 J/g. The differences observed between the denaturation parameters of bovine pericardium and pure collagen type I extracted from bovine tendon can be explained by the arguments previously given in the hydrated state.

The thermal characteristic of the valvular leaflet also shows strong analogies with the collagen and pericardium thermograms: an endothermic peak associated with collagen denaturation is observed at Td = 232.2 °C with ΔHd = 10.3 J/g. Considering that the valvular leaflet contains 70% of collagen and only 6% of elastin, it is coherent to observe only the thermal signature of collagen in this kind of tissues. A previous work showed a strong analogy between the thermograms of human valvular leaflet and porcine valvular leaflet, confirming the ability of porcine bioprostheses to replace human aortic valves at a molecular level [35,36].

Finally, the thermogram of aortic wall is the more complex one: an endothermic event associated with the collagen denaturation is evidenced at Td = 234.2 °C with ΔHd = 3.9 J/g. It is worth noting that the value of denaturation enthalpy value is roughly proportional to the percentage of collagen in the studied tissue (3.9 J/g for the aortic wall, containing 23% of collagen, and 10.3 J/g for the valvular leaflet, containing 70% of collagen). The thermal signature of elastin is also evidenced as a step of the specific heat at 204.7 °C.

The assignation of thermal transitions can be performed in control cardiovascular tissues, in the hydrated and dehydrated states. The evolution of these parameters can be used to check the integrity of collagen and elastin in biomaterials; on this basis three applications are detailed in the following sections.

### 2.2. First Application: Classification of Chemical Treatments for the Stabilization of Collagen in Bovine Pericardium

**Figure 3:**
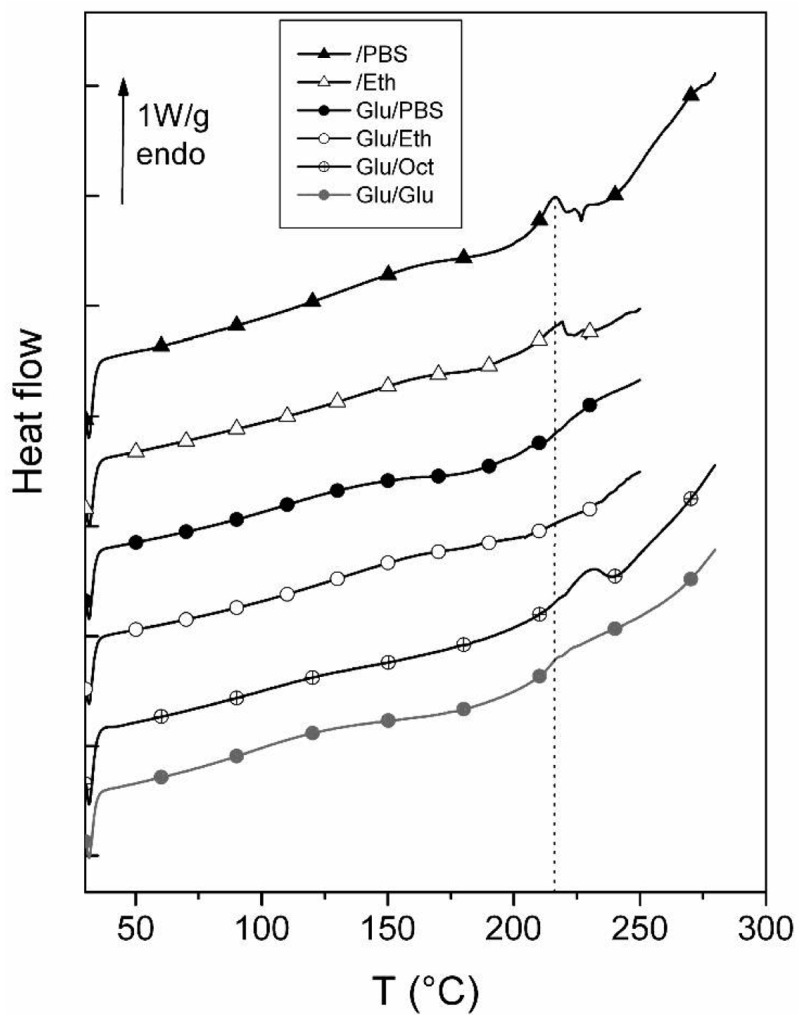
DSC thermograms of bovine pericardium samples in the dehydrated state recorded at 20 °C/min from 30 to 250 or 280 °C.

In this application we focus on the characterization of collagen in acellular bovine cardiovascular tissues, fresh or glutaraldehyde treated, and stored in different solutions (phosphate buffered saline (PBS), ethanol, octanol and gluateraldehyde), to determine whether the resulting fibrous material is structurally preserved. Figure 3 shows the DSC second thermograms of the differently treated and freeze-dried samples.

The thermal events occurring in the 180–280 °C zone, namely the denaturation window, is the zone of interest in this study. The denaturation peak of the control sample, stored in PBS, is quite similar to the endothermic denaturation of pure collagen. It is noteworthy that the glutaraldehyde treated sample, stored in octanol, possesses a well-defined endothermic denaturation that can be compared, for the general shape, with the control sample stored in PBS. In Table 1 we reported the corresponding denaturation temperatures Td computed from a statistical study.

**Table 1:** Denaturation parameters of the bovine pericardium samples possessing a well-defined denaturation peak.

	***Td (°C)***	***Hd (J/g)***
***\PBS***	214.8 ± 0.8	12.3 ± 2.4
***Glu\Oct***	230.0 ± 0.6	7.8 ± 0.7

Significant difference on Td mean values: p = 1.13 × 10^−4^; Significant difference on ΔHd values: p = 4.23 × 10^−9^.

The denaturation temperature is shifted toward high temperature with glutaraldehyde. As for the other samples, we did not detect the well-shaped denaturation peak expected in this zone of temperature. A step of the specific heat can be detected on Glu/PBS and Glu/Glu samples, certainly associated with the glass transition of gelatin.

The study of the thermal denaturation of collagen in differently treated bovine pericardium samples provides us with interesting information on the effects of treatment on the triple helical structure and stability of this protein. The control sample possesses a well-defined endothermic denaturation at 215 °C, as is generally expected for lyophilized collagenic material. Another tissue presents a well-shaped denaturation, namely the glutaraldehyde sample stored in octanol. In this case, the denaturation temperature is shifted toward high values, allowing us to conclude that the triple helical domains of collagen are preserved and above all, stabilized by glutaraldehyde and preserved by octanol storage. This result is in good agreement with several studies that showed the stabilizing effect of glutaraldehyde on biological tissues [5,6]. The cross-linking action of this product can explain this peculiar behavior [3,5,7]. That is the reason that glutaraldehyde is used in many graft procedures, stabilizing and preventing proteins from degradation.

All the other treatments have a harmful influence on the preservation of triple helical structure. Storage in ethanol of the untreated sample seems to change the triple helical structure of collagen, increasing the heterogeneity (multiple peaks) and facilitating the uncoiling of the protein (weak denaturation enthalpy). This assumption must be associated with the texture of the air-dried sample (strong rigidity) that does not have the features of the freeze-dried sample stored in PBS; all the samples treated with this procedure have this peculiar feature. The denaturation of the sample treated with glutaraldehyde and stored in PBS is very weak and broad, and the denaturation, hardly detectable, is shifted toward high temperature. We can conclude that the denaturation phenomenon is impaired by the combination of glutaraldehyde treatment and storage in PBS buffer.

The combination of glutaraldehyde treatment and ethanol storage involves drastic alterations of the helical structure of collagen. The fraction of denatured collagen is certainly important. This aspect of the air-dried sample (modification of color and rigidity, from all the series) is also indicative of profound chemical and/or structural modifications.

As for the sample treated with glutaraldehyde and stored in the glutaraldehyde solution, the denaturation phenomenon is not well defined; it reveals a narrow peak or a very weak peak. Some triple helical domains must subsist, differently cross-linked. The heterogeneity and the fraction of denatured collagen are important.

This work on bovine pericardium samples has allowed to propose a classification of different chemical treatments and storage conditions: the best conditions for the preservation of collagen structure and the enhancement of triple helical stabilization are the glutaraldehyde treatment followed by octanol storage; this procedure must be now applied to the conception of bioprosthetic heart valve, in order to increase the allografts' durability *in vivo.*

### 2.3. Second Application: Effect of Low Temperature Plasma Jet on Thermal Stability of Type I Collagen

Non-thermal and low-temperature plasmas generated at atmospheric pressure have been extensively used in various biomedical applications such as sterilization and decontamination [37,38], tissue engineering and biomaterial treatment for their functionalization [39,40]. More recently they have been used in plasma medicine for blood coagulation, disinfection of living tissues involving wound healing and, more generally, for the interaction of plasmas with eukaryotic cells [41,42,43]. The non-thermal and low-temperature plasmas of our interest are generated at atmospheric pressure by corona or dielectric barrier discharges under specific geometry of electrode configuration. In the present work, for the collagen treatment we used a non-thermal and low-temperature plasma jet generated in ambient air and producing various active species that are in contact with collagen fibers during the remote plasma treatment.

#### 2.3.1. Dehydrated State

**Figure 4:**
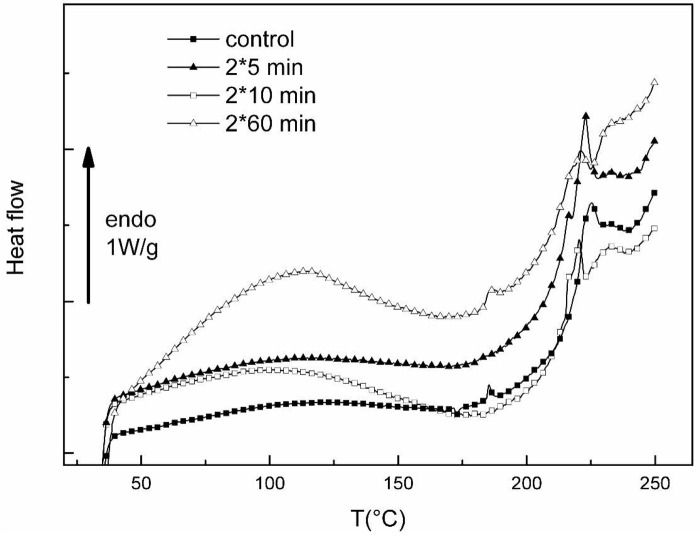
DSC thermograms of freeze-dried collagens (control and plasma jet treated collagens) recorded at 20 °C/min between 40 and 250 °C.

Figure 4 shows the DSC thermograms of control collagen and collagen treated by the plasma jet for several time exposures in the freeze-dried state.

The thermal events occurring in the 180–280 °C range are more particularly focused. Table 2 displays the corresponding denaturation temperatures Td (defined as the maximum of the peak) and the corresponding enthalpies of denaturation ΔHd computed from a series of measurements.

**Table 2:** Denaturation parameters of freeze-dried collagens (control and plasma jet treated).

***Sample***	***ΔHd (J/g)***	***Td (°C)***
***Control***	7.05	225
***5 min***	13.7	215–223
***10 min***	8.42	215–220–230
***60 min***	3.7	217–220–231

The denaturation parameters of control freeze-dried collagen Td =225 °C and ΔHd = 7.05 J/g were discussed in section 2.1.2.

With the plasma exposure, several changes are noted on the denaturation event: multiple peaks appear even for 5 min exposure. The denaturation becomes a complex event, implying zones that denature at lower temperature (Td = 215 °C), with a large denaturation enthalpy. For a 10 min exposure, there are denaturing zones at temperatures Td = 215 °C and 220 °C, and denaturing zones at a higher temperature (Td = 230 °C), the total denaturation enthalpy being similar to control collagen one. For a 60 min exposure, the different zones of denaturation are still present on thermograms, but in this case the total enthalpy of denaturation is drastically decreased. Previous research on type I collagen irradiated by UVB [24] has reported the existence of denaturation peaks shifting towards low temperature and higher temperature, and attributed to destabilized zones and stabilized zones, respectively. By comparison with data on differently treated collagens, the destabilized zones could be attributed to the reduction of collagen in polypeptides of different molecular weight [24]. Nevertheless, the high enthalpy associated with destabilized zones for the 5 min exposure is a feature of a triple helical domain. A similar phenomenon was observed by calorimetric experiments for short time exposure to UVC (λ = 254 nm) and it was addressed to an intermediate state before degradation or cross-linking. The stabilized zones can be attributed to a cross-linking of collagen; for 10 min exposure, the total enthalpy is slightly greater than the enthalpy corresponding to control collagen, meaning that the stabilization is mainly entropic, such as induced by an increase in the packing density of collagen molecules in the fibers. Such a phenomenon was reported both with UVB [24] and UVC [44] irradiation or with the presence of a photosensitizing agent and laser irradiation in the visible spectra [45]. As the low temperature plasma jet generates radicals, excited and ionized species and also UV emissions, cross-linking of collagen in our experiments could be partly attributed to the UV emissions of plasma jet. However, this affirmation must be considered with care because it has to be confirmed by a plasma jet treatment of the collagen done with and without an UV filter. It must be recalled that collagen type I contains phenylalanine and tyrosine, aromatic residues that are the main absorber of UV light, and that are shown to decrease with UV irradiation, due to a possible aggregation of the fibers [46]. For a 60 min irradiation, the shape of denaturation peaks and the low value of the associated enthalpy suggest an important degradation of collagen triple helical structure. The optimum time seems to be between 10 and 60 min.

As observed in the case of UV irradiation, with the increasing exposure time to the low temperature plasma jet, peptide bond cleavage becomes predominant. The relative proportion of these two competing reactions, both cross-linking and bond cleavage is unknown but was shown to depend on the water content and the oxygen tension [24]. That is the reason for which similar experiments were also performed on hydrated collagen.

#### 2.3.2. Hydrated State

**Figure 5:**
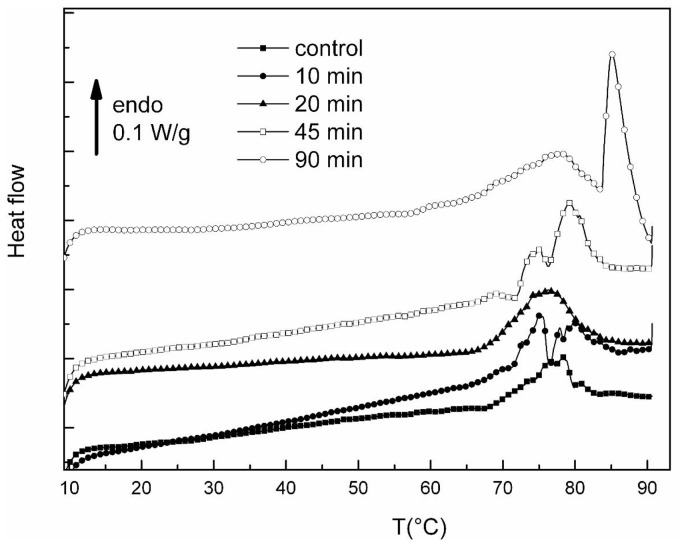
DSC thermograms of hydrated collagens (control and plasma jet treated collagens recorded at 10 °C/min between 10 and 90 °C.

Figure 5 displays the DSC thermograms of control hydrated collagen (*i.e.*, without plasma treatment) and hydrated collagen treated by the plasma jet for gradual time exposures (up to 90 min).

Some authors [23] showed that the denaturation enthalpy of collagen was constant above 6 moles of water per tripeptide of collagen (corresponding to 0.4 g of water/g collagen), and that the denaturation enthalpy was constant above 30 moles of water per tripeptide (corresponding to 1.9 g of water per g of collagen). In the present study, the levels of hydration were checked to be largely superior to these limits, allowing us to compare the denaturation parameters with time without fluctuations due to distinct hydration.

A complex endothermic peak addressed to the denaturation phenomenon of hydrated collagen is detected for all the samples, splitting into two components or more for 10 min, 45 min and 90 min of plasma exposure times. Table 3 displays the corresponding denaturation temperatures Td and the corresponding enthalpies of denaturation ΔHd computed from a series of measurements.

**Table 3:** Denaturation parameters of hydrated collagens (control and plasma jet treated).

**Sample**	**ΔHd (J/g dry collagen)**	**Td (°C)**
**Control**	47.8	78.3
**10 min**	66.4	75.1–79.9
**20 min**	48.8	76.5
**45 min**	70.6	75.2–79.1
**90 min**	126.2	78.0–85.1

The denaturation parameters of control hydrated collagen (Td = 78.3 °C and ΔHd = 47.8 J/g) were discussed in section 2.1.1.

For an exposure time of 10 min, the split of the denaturation peak can be attributed to destabilized and stabilized zones, showing in this case, as in the dehydrated one, the competition between two antagonist processes: reduction into polypeptides of various molecular weights and cross-linking of collagen. The value of enthalpy indicates a conservation of the triple helical structure. Nevertheless, a qualitative comparison between the two peaks shows that the destabilization mechanism is dominant. For an exposure time of 20 min, stabilized zones and destabilized zones must be roughly formed in equal parts, giving rise to a broad and complex denaturation peak. For a 45 min exposure, the cross-linking mechanism appears to be the main phenomenon according to peak height and area. Finally, a special feature is noticed for an exposure time of 90 min. Contrary to the evolution at long exposure time previously reported in the dehydrated case, with a drastic decrease of the enthalpy, in this hydrated case a sharp and intense peak is detected at 231 °C, corresponding to a highly stabilized collagen domain. The peak previously attributed to destabilized zones is also shifted towards higher temperature, the temperature of the peak maximum being analogous to the control collagen one. So the cross-linking mechanism is rather distinct from that observed in the dehydrated sate, and seems better to obtain highly stabilized samples.

The present work of the thermal denaturation of collagen in differently treated collagen samples presents us with interesting information on the effects of the low temperature plasma jet treatment on the triple helical structure and the stability of this protein. There is a competition between reduction of collagen in polypeptides and cross-linking mechanism. In the freeze-dried state, the destabilization of the triple helical structure is the main event for the longest exposure times. The feature is distinct when collagen I fibers are exposed to the plasma jet in the hydrated state. In this case, the cross-linking phenomenon begins predominantly for the longest exposure times (between 45 min and 90 min). Therefore, it is clear that the plasma jet treatment of freeze-dried fibers must be avoided.

Anyway, the present exploratory work is a first step to determine the best conditions for the preservation of the collagen structure and the enhancement of triple helical stabilization. However, as plasma jet produces many active species (radicals, excited and ionized species and also photon emissions from UV up to near Infra-Red range), future experiments will be done in order to identify the most active species by using, for instance, filters for UV emissions and charged particle impacts.

Furthermore, the present plasma treatment is first applied to collagen type I and pericardium. So, it will be interesting to extend this procedure to other biomaterial tissues to provide interesting fibrous material for the conception of bioprosthetic materials. Lastly, it will be also very interesting to study the biocompatibility and the cellular adhesion and proliferation of the collagenous biomaterials exposed to the low temperature plasma jet.

### 2.4. Third Application: Comparison of Detergent Treatments for Cardiac Valves Bioprostheses

One major problem is the elaboration of the most appropriate treatment for removal of cell debris and maintenance of the structural integrity of the collagen/elastin matrix. In this study, two multi-step extractions (TRI-COL and SDS extractions) have been achieved on porcine aortic tissues in order to obtain acellular matrices as used for cardiac bioprostheses [47]. The evaluation of structural modifications and possible damages on extracellular matrix fibrous proteins has been investigated by means of DSC.

**Figure 6:**
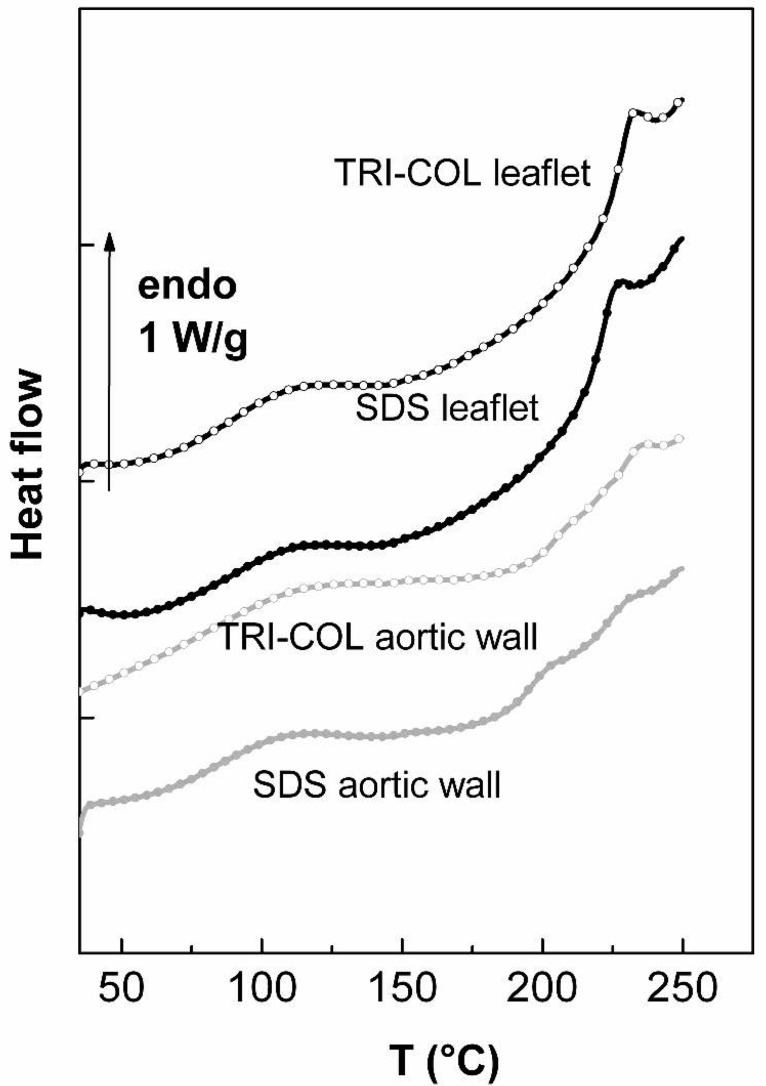
DSC thermograms of freeze-dried porcine aortic tissues (TRI-COL and SDS treated tissues) recorded between 40 and 250 °C recorded after a first scan performed between 40 and 150 °C.

Since preliminary DSC scans are characteristic of proteins-water interactions, few differences between differently treated tissues have been noted. In this investigation, we focus on the evolution of the protein transitions of aortic wall and valvular leaflet following treatment with SDS. To precisely determine the influence of the detergent treatment on the proteins physical structures, second thermograms corresponding to the TRI-COL and SDS treated aortic tissues have been reported in figure 6.

#### 2.4.1. Valvular Leaflet

The comparison between the two scans clearly shows a shift of the collagen denaturation temperature towards the low values for the SDS treated leaflets (from 232 °C to 225 °C). A statistical analysis performed on a series of measurements confirms the significance of the results (p < 0.00005, see table 4); on the other hand, the denaturation enthalpy seems also to decrease with SDS treatment but analysis of values shows that it is not reduced in a significant manner (p > 0.05).

**Table 4:** Transition parameters from DSC (second scans) for differently treated leaflets and aortic walls.

	***collagen***		***elastin***
	***ΔH_d_ denaturation (J/g)***	***T_d_ denaturation (°C)***	***T_g_elastin (°C)***
***TRI-COL leaflet***	10.33 ± 0.54	232.3 ±0.4	Not meas.
***TRI-COL aortic wall***	3.89 ± 0.33	234.2 ±0.5	204.7 ± 0.4
***SDS leaflet***	11.65 ± 0.4	224.9 ± 0.7	Not meas.
***SDS aortic wall***	3.04 ± 0.38	228.6 ± 0.9	196.7 ± 0.5

#### 2.4.2. Aortic Wall

In a similar way to that previously described, the influence of SDS extraction on the aortic wall phase transitions has been studied by the comparison of the DSC thermograms. On one hand, the first order transition associated with collagen denaturation undergoes the same evolution as previously shown in the case of leaflet: with reference to the TRI-COL treatment the temperature transition is lowered from 234 °C to 229 °C after SDS treatment (p < 0.005), while the denaturation enthalpy is not subjected to significant changes. On the other hand, a significant decrease of the elastin temperature glass transition with SDS (from 205 °C to 197 °C, p < 0.005) is noted.

The DSC technique is well suited to reveal the effects of SDS on the proteins structures; indeed the comparison between the differently treated tissues thermograms shows that SDS acts on all intrinsic transitions in a similar manner, *i.e.*, a shift toward the low temperature values. The decrease of collagen temperature denaturation after SDS treatment has been observed previously in solution either by DSC technique or shrinkage [48,49] and had been interpreted as a loss of collagen thermal stability. The SDS treatment does not denature collagen in the sense that triple helical domains are conserved, but, as shown by transmission electron microscopy, the quarter-stagger conformation seems less stable [12]. The lowering of elastin glass transition temperature with SDS is in accordance with previous works on SDS/elastin interactions: SDS is an anionic hydrophobic ligand whom release rates from fibrous elastin on repeated washing with an SDS free buffer are much smaller than those from powder elastin [50] and therefore could be residual in the extracted samples. This component could alter the mechanical properties of elastin either by binding to the polypeptidic chains within the fibers [51,52] or rather, as it was suggested [50], by binding to the interfibers spaces and outer surfaces of the fibers.

In this investigation, the previously reported denaturing action of SDS on collagen fibers has been correlated to changes of specific structural parameters in comparison with TRI-COL treatment which appeared to be devoid of such evident destabilizing effects and to preserve the structural integrity of collagen and elastin network in both valvular leaflet and aortic wall.

## 3. Experimental Section

### 3.1. Preparation of the Samples

#### 3.1.1. Bovine Pericardia

Six types of tissues were prepared using different treatments and storage conditions as detailed below.

- Control samples are fresh tissue stored in PBS: bovine pericardium was obtained fresh from the abattoir and placed in chilled phosphate buffered saline (pH 7.4) until the time of analysis or implantation. Extraneous fat or muscle was removed and sections with heavy vasculature or attached ligaments were discarded. These samples are referred to as control or /**PBS**.
- *Fresh tissue stored in 80% ethanol:* fresh bovine pericardium stored in chilled PBS was transferred to 80% ethanol buffered HEPES (10mM) solution for 3 days prior to analysis. These samples are referred as /**Eth**
- *Glutaraldehyde treated, PBS stored:* fresh bovine pericardium stored in PBS was transferred to a buffered solution of 0.25% glutaraldehyde for 1 week. The tissue was transferred to chilled PBS for 3 days prior to analysis. These samples are referred as **Glu/PBS**.
- *Glutaraldehyde treated, stored in 80% ethanol:* glutaraldehyde treated bovine pericardium was transferred to 80% ethanol buffered HEPES (10mM) solution for 3 days prior to analysis. These samples are referred as **Glu/Eth**.
- *Glutaraldehyde treated, stored in octanol:* glutaraldehyde treated bovine pericardium was transferred to 5% octanol/40% ethanol solution for 3 days prior to analysis. These samples are referred as **Glu/Oct**
- *Glutaraldehyde treated, stored in glutaraldehyde:* fresh bovine pericardium stored in PBS was transferred to a buffered solution of 0.25% glutaraldehyde for 1 week prior to analysis. These samples are referred as **Glu/Glu**.

Prior to DSC and TSC experiments, each of the tissues were flash frozen in liquid nitrogen and freeze-dried in a Freezone^®^ 4.5 freeze dry system (Labconco Corp., Kansas City, MO, USA).

#### 3.1.2. Collagen Treated by the Plasma Jet

Commercial insoluble type I collagen (Fluka Chemie AG, Switzerland) was extracted from bovine Achilles tendon and available in the form of air-dried fibers.

The low temperature plasma jet used in the present work for the collagen treatment has been the subject of a patent [53]. The measured plasma temperature on the top of the jet, that has a length of about 1 cm, does not exceed 27 °C. The plasma jet is generated directly in the ambient air at atmospheric pressure and launched by itself without any system of gas inlet feed that making it very easily transportable because there is neither gas bottle nor gas pumping. It is a low-temperature plasma generated by a specific corona discharge design giving a natural repetitive discharge current with a frequency of about 20 kHz under a high voltage power supply.

##### Treatment in the freeze-dried state

Collagen fibers were compressed into pellets (thickness 0.5 mm, diameter 10 mm) and each face of the pellet was exposed to the plasma jet during 5, 10 and 60 min at ambient temperature (20 °C).

##### Treatment in the hydrated state

80 mg of collagen fibers were placed in 7 mL of deionized water, equilibrated for 1 hour so that the fibers were swollen and subsequently exposed to the plasma jet during 10, 20, 45 and 90 min under stirring at ambient temperature (20 °C). A set of samples were then carefully put down absorbent paper to remove excess water before thermal analysis. Another set of samples were freeze-dried again before further thermal analysis.

Bovine pericardium was obtained fresh from the abattoir and placed in chilled phosphate buffered saline (pH 7.4) until the time of analysis. Extraneous fat or muscle was removed and sections with heavy vasculature or attached ligaments were discarded. Then, 80 mg of hydrated bovine pericardium (strips 1 mm × 10 mm × 10 mm) were exposed to the plasma jet during 20 and 45 min. Thermal analysis was performed immediately after treatment.

#### 3.1.3. Porcine Valvular Leaflets and Porcine Aortic Walls

Aortic roots comprising terminal part of the aortic wall, sinus valvularis and corresponding leaflets were freshly dissected from the heart of young pigs. One set of such samples was suspended in a degassed physiological buffer (50 mM HEPES, 0.1 M NaCl, pH 7.4) containing protease inhibitors (5 mM EDTA, 2 mM PMSF, 5 mM NEM, 5 mM Benzamidine, 1 mM Iodoacetamide), 10 mM sodium ascorbate and 10% DMSO. The surrounding solution was gently stirred at 4 °C for 3 hours under N_2_ atmosphere. Then samples were extracted in the same conditions by replacing DMSO with 1% (w/w) SDS at 37 °C for 16 h and for further 3 × 16 h periods in the absence of protease inhibitors.

Another set of samples was treated initially in the same conditions as the first set but replacing the physiological buffer with hypotonic, 10 times diluted, PBS buffer pH 7.4. Then DMSO was replaced with 1% (w/w) Triton X-100 and samples were extracted for 10 h at 4 °C in the same conditions. After a further 10 h extraction in the absence of protease inhibitors, Triton X-100 was replaced by 10 mM Sodium Cholate and further extracted for 2 × 10 h periods at room temperature. Samples treated with Triton and Cholate as above will be further referred to as TRI-COL samples.

Both sets of samples were then washed with 10% isopropanol, first in saline water and second in saline and deionized water before freeze-drying and drying under reduced pressure over P_2_O_5_.

### 3.2. Differential Scanning Calorimetry (DSC)

The phase transition thermograms were recorded with a DSC7 differential scanning calorimeter from Perkin Elmer. The temperature and energy scales were calibrated using the manufacturer's instructions with Mercury, Indium and Tin as standards. Samples of 5 mg of weight were sealed in aluminum pans. Empty pans were used as references. Thermal analysis is mainly performed to get insight into the denaturation phenomenon of collagen, which is known to occur in the 40–80 °C in the hydrated state and between 180 and 230 °C in the dehydrated state. That is the reason why investigations in the hydrated state were performed between −40 and 90 °C with 10 °C/min heating rates, in hermetic pans. Investigations in the dehydrated state were performed between 30 °C and 250 °C with 20 °C/min heating rates, in non-hermetic pans. Determination of transition parameters was performed with Origin software.

## 4. Conclusions

This work shows the ability of Differential Scanning Calorimetry to check the integrity of elastin and collagen in different kinds of biomaterials analyzed in hydrated conditions, physiological conditions, or in the freeze-dried state, avoiding the extrinsic response of water. The thermal stability of the triple helical domains of collagen, an ordered biopolymer, is directly related to the denaturation parameters—temperature and enthalpy of denaturation—that can be compared in the different biomaterials, allowing a classification of the different treatment of cellular extraction or collagen stabilization. The physical structure of elastin, an amorphous biopolymer, is connected to its glass transition. The comparison between the values of the glass transition temperature can reveal differences in the elastin network and allows predicting the mechanical properties. This work will be completed in a following paper by dielectric analyses (thermally stimulated depolarization currents-TSDC) performed on the biomaterials studied here. Dielectric techniques give more detailed information on the molecular dynamics and chain dynamics of collagen and elastin, more particularly on localized motions (secondary relaxations) and on amorphous phase.
